# The robustness of multiplex networks under layer node-based attack

**DOI:** 10.1038/srep24304

**Published:** 2016-04-14

**Authors:** Da-wei Zhao, Lian-hai Wang, Yong-feng Zhi, Jun Zhang, Zhen Wang

**Affiliations:** 1 Shandong Provincial Key Laboratory of Computer Networks, Shandong Computer Science Center (National Supercomputer Center in Jinan), Jinan 250014, China; 2 School of Automation, Northwestern Polytechnical University, Xian 710072, China; 3 Interdisciplinary Graduate School of Engineering Sciences, Kyushu University, Kasuga-koen, Kasuga-shi, Fukuoka 816-8580, Japan

## Abstract

From transportation networks to complex infrastructures, and to social and economic networks, a large variety of systems can be described in terms of multiplex networks formed by a set of nodes interacting through different network layers. Network robustness, as one of the most successful application areas of complex networks, has attracted great interest in a myriad of research realms. In this regard, how multiplex networks respond to potential attack is still an open issue. Here we study the robustness of multiplex networks under layer node-based random or targeted attack, which means that nodes just suffer attacks in a given layer yet no additional influence to their connections beyond this layer. A theoretical analysis framework is proposed to calculate the critical threshold and the size of giant component of multiplex networks when nodes are removed randomly or intentionally. Via numerous simulations, it is unveiled that the theoretical method can accurately predict the threshold and the size of giant component, irrespective of attack strategies. Moreover, we also compare the robustness of multiplex networks under multiplex node-based attack and layer node-based attack, and find that layer node-based attack makes multiplex networks more vulnerable, regardless of average degree and underlying topology.

Robustness of networks refers to the ability of preserving their functional integration when they are subject to failures or attacks^1^,^2^. Understanding the robustness of networks is thus useful for evaluating the resilience of systems and constructing more efficient architectures. During the past decades, there have been a great number of works contributing to this topic. But the majority of these achievements mainly focus on the vulnerability of single-layer networks^3^,^4^,^5^,^6^,^7^,^8^,^9^, which seems inconsistent with the well-recognized fact that nodes can simultaneously be the elements of more than one network in most, yet not all, natural and social systems^10^,^11^,^12^?. Recently, Buldyrev *et al.* studied the robustness of interdependent networks, where two networks were coupled in one-to-one interdependence way^13^. Following the failure of one node, a cascading crash took place in both networks (namely, interdependent networks are intrinsically more fragile than traditional single-layer networks), which was accurately validated by the theoretical analysis as well. After this interesting finding, the research of network science is fast extended to multilayer framework^14^,^15^,^16^,^17^, where systems are usually composed of several network layers, including interdependent networks^18^,^19^,^20^,^21^,^22^,^23^,^24^,^25^,^26^, interconnected networks^27^,^28^,^29^,^30^,^31^,^32^ and multiplex networks^33^,^34^,^35^,^36^,^37^,^38^,^39^,^40^,^41^,^42^,^43^,^44^. Thus far, the topological characteristics of multilayer networks and dynamical process (such as evolutionary game theory^22^,^24^, disease spreading^28^,^31^,^37^,^43^,^45^, random diffusion^33^ and synchronization^39^) upon them have attracted great attention in both theoretical and empirical areas (for a recent review see^14^).

Different from interdependent netowrks, multiplex networks, as a typical kind of topology structures, can be regarded as the combination of several network layers which contain the same nodes yet different intra-layer connections. In this sense, many real-world systems like online social networks^46^, technological networks^47^, transportation networks^48^ can be further studied with the viewpoint of multiplex networks. Figure 1 gives an illustration of multiplex framework: six people are connected via two kinds of relationship, for example Facebook connections (blue links) and Twitter connections (black links) (panel (a)). Such systems can be well embedded into the framework of multiplex networks with two types of links. Each link type in the system defines a network layer, and the nodes of each network layer are the same (see panel (b)). To distinguish the node of multiplex networks and its replica in each network layer, we term them as multiplex node and layer node, respectively. The former points to the node which connects its neighbors via all the types of links, like node 3 of Fig. 1(a), whose neighbors are 1, 5 and 4 via blue and black links. While the latter is the partial case of multiplex node and just considers the local connection topology of a given node on one layer. For example, if we only consider blue (black) links in Fig. 1(b), node 3 is the layer node of Layer-1 (Layer-2). Now, it is thus clear that multiplex node means the joint element of all the layers, and layer node just belongs to the element of one given layer.

Looking back to the early topic, the research of robustness of multiplex networks thus becomes a very interesting and crucial challenge. In^44^, Min *et al.* explored the robustness of multiplex networks when multiplex nodes were removed randomly or intentionally (here the removal of a multiplex node means all its replicas in network layers are pruned). They showed that correlated coupling would affect the structural robustness of multiplex networks in diverse fashion. In some realistic cases, however, the failure unites or attack targets may be just the layer nodes. For example, on multiplex transport networks where nodes are cities and network layers are airplane network, highway network and railway network, the failures may take place in one or some, yet not all the layers. Similarly, prohibiting the use of some social network accounts, people may still connect with each other via other available social network accounts. In this sense, an interesting question naturally poses itself, which we aim to address in this work. Namely, how does the removal of layer node affect the robustness of multiplex networks?

Aiming to answer this issue, we consider the robustness of multiplex networks under layer node-based attack, which can be further divided into random and targeted scenarios. With the framework of generating function method^49^, we propose theoretical method to calculate the critical threshold of network crash and the size of giant component when a fraction of layer nodes are removed. Furthermore, we also compare the robustness of multiplex networks under multiplex nodes-based attack and layer node-based attack.

## Results

### Model definition and theoretical analysis

**Figure 2:**
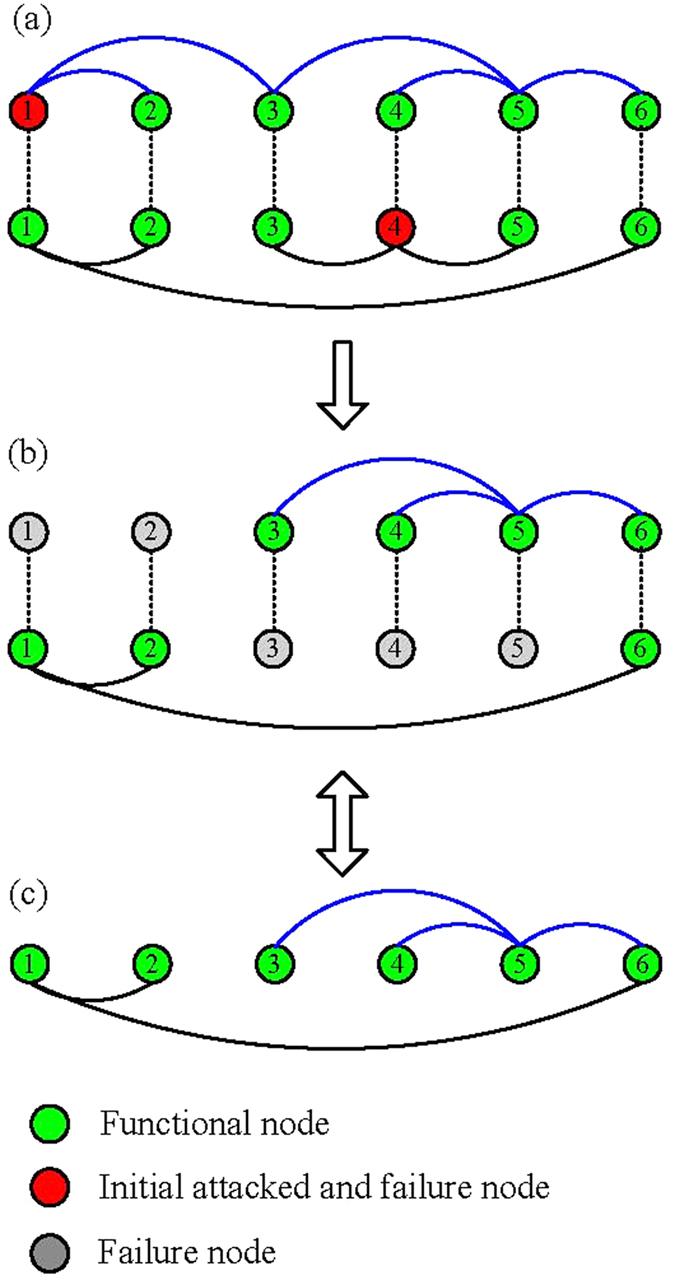
Schematic illustration of layer node-based attack and giant component of multiplex networks. (**a**) Layer node 1 of Layer-1 and layer node 4 of Layer-2 are initially attacked. (**b**) Soon layer nodes 1,2 of Layer-1 and 3,4 and 5 of Layer-2 become failure nodes since they do not belong to the giant component of corresponding layers. (**c**) However, multiplex nodes 1–6 still belong to the mutually connected giant component of the multiplex networks since they connect to the largest component through at least one type of links after damage.

As mentioned in previous literatures^13^,^21^,^23^, the robustness of networks is usually evaluated by one critical threshold value and the size of giant component after the removal of nodes. If the fraction of removed nodes exceeds this critical threshold, the giant component becomes null. Here it is worth mentioning that the accurate definition of giant component of multiplex networks should be mutually connected giant component (MCGC), which is the largest component that remains after the removal propagates back and forth in the different layers. The giant component naturally consists of a set of connected multiplex nodes. A pair of multiplex nodes are regarded to have connection if there exists at least one type of link between them. Therefore, attacking some layer nodes may not destroy their connection with other nodes, and the set of nodes that remains at the end of damage is the mutually connected giant component (see Fig. 2 for schematic illustration). In the following, we will focus on theoretical method of calculating the critical threshold value and the size of giant component of the multiplex networks under layer node-based attack.

For simplicity (yet without loss of generality), we refer to previous treatment^2^,^44^,^49^. For a multiplex network composing of *N* multiplex nodes and *m* network layers, the generating function for the joint degree distribution 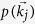, where 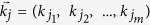 denotes the degrees of a multiplex node *j* in each layer, can be written in the form of a finite polynomial


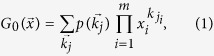


where 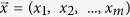 represents the auxiliary variable coupled to 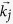. Here, it is of particular value to emphasize give that the key parameter, joint degree distribution 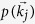, contains the general information of degree correlation between network layers, the following derivations will be universal for (un)correlated multiplex networks. Then the generating function of remaining joint degree distribution by following a randomly chosen link of network layer *i* is given by


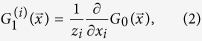


where *z*_*i*_ is the average degree of layer *i*.

Then, on locally-tree like networks, if *u*_*i*_ (*i* = 1, 2, …, *m*) is defined as the probability that a multiplex node reached by following a random chosen link of network layer *i* does not belong to the giant component, it can be derived by the coupled self-consistency equation


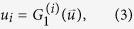


where 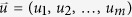. Furthermore, the size of the (mutually connected) giant component can be calculated according to


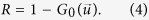


Along this framework, we can now turn to the layer node-based attack on multiplex networks. If 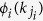 is used to denote the probability that a layer node with degree 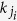 is removed from network layer *i*, then the generating function of the joint degree distribution after the removal of layer nodes can be expressed as


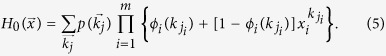


Correspondingly, the generating function of remaining joint degree distribution after the removal of layer nodes by following a randomly chosen link of network layer *i* is given by


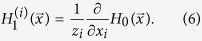


In the case of layer node removal, the probability *v*_*i*_ that a multiplex node reached by following one random chosen link of network layer *i* does not belong to the giant component can be written as


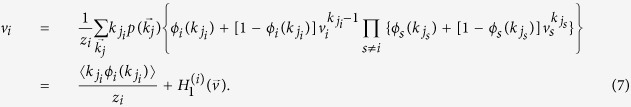


Then, after the removal of nodes from layers, the size of giant component is given as follows


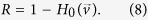


The existence of giant component under layer node-based attack requires the largest eigenvalue Λ of the Jacobian matrix **J** of Eq. (7) at (1, 1, …, 1) to be larger than unity^44^. In this work, we mainly focus on multiplex networks composed of two Erdös-Rényi (ER) random^50^ or Barabási-Albert scale-free (SF)^51^ network layers (namely, *m* = 2), **J** thus can be written as


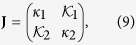


where 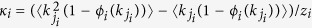 and 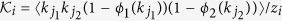. The largest eigenvalue Λ is given by


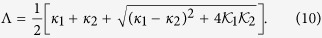


Before computation simulations, it is worth mentioning that the above derived theoretical framework (of generating function) is even effective in the thermodynamic limit *N* → ∞. Aiming to validate its accuracy, we will pay our main attention to middle-size networks in the following simulations.

### Layer node-based random attack

For layer node-based random attack, which is characterized by random removal of layer nodes from network layers, there exists the removal probability 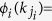 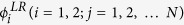. According to the above analysis, the critical threshold and the size of giant component of multiplex networks under layer node-based random removal can be respectively expressed as


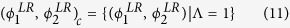


and


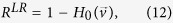


where 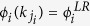. It is worth mentioning that above 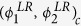 there is no giant component, whereas below 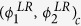 a giant connected cluster exists.

**Figure 3:**
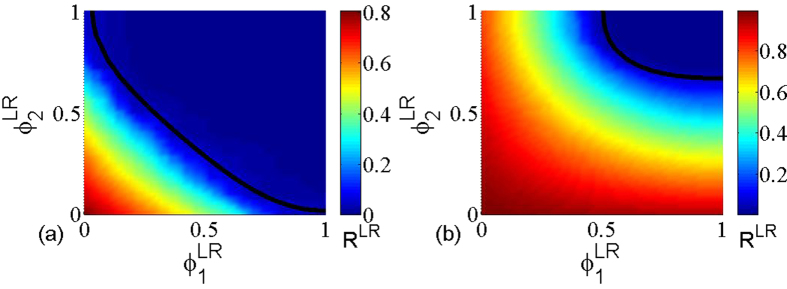
The size *R*^*LR*^ of giant component in dependence on removal probability 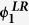 and 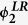 for layer node-based random attack. The black line indicates the theoretical critical threshold calculated according to Eq. (11). The networks used are multiplex ER network with average degree (**a**) *z*_1_ = *z*_2_ = 1, (**b**) *z*_1_ = 2, *z*_2_ = 3 and network size *N* = 5000. It is worth mentioning that the well agreement between theoretical prediction and simulation outcome is effective for larger networks as well (not shown here). For the convenience of simulations, we will focus on the same network size in the remaining figs.

**Figure 4:**
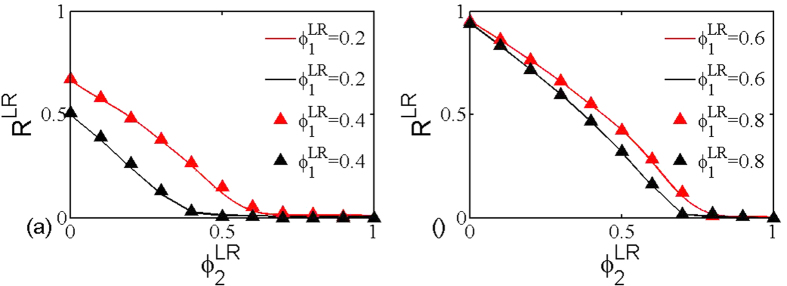
Theoretical (line) and numerical (point) results of the size *R*^*LR*^ of giant component as a function of 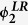 when 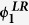 takes fixed values. The networks used are multiplex ER networks with average degree (**a**) *z*_1_ = *z*_2_ = 1, (**b**) *z*_1_ = 2, *z*_2_ = 3 and network size *N* = 5000.

We start by inspecting how layer node-based random attack affects the robustness of multiplex networks. Figure 3 shows the size *R*^*LR*^ of giant component in dependence on the removal probability 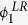 and 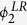 for network layer 1 and network layer 2, respectively. Moreover, the black line indicates the theoretical critical threshold calculated according to Eq. (11). It is clear that when the removal probability (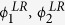) is above this black line, the size of giant component becomes negligible; whereas there exists one giant component if (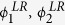) is located below this black line. This implies that the theoretical critical threshold can accurately predict the impact of layer node-based attack on the robustness of multiplex networks. To further validate this fact, we also compare the theoretical prediction derived from Eq. (12) and simulation results for the size of giant component in Fig. 4. It can be observed that there is indeed good agreement between simulation and theoretical prediction.

### Layer node-based targeted attack

Targeted attack, as a well-known attack strategy, usually aims to remove influential nodes, which can be identified by centrality measures, such as the degree centrality, eigenvector centrality, *k*-shell centrality and betweenness centrality^52^. In this work, we mainly pay attention to the viewpoint of degree centrality. For layer node-based targeted attack, the removal probability of a layer node with degree 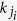 is determined by its degree, and can be expressed as follows


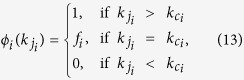


where 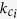 is the cutoff degree for attack on network layer *i*, and *f*_*i*_ denotes the removal probability of node with degree 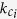. Consequently, the total fraction of removal nodes in network layer *i* is given by


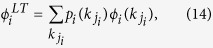


where 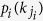 indicates the fraction of layer nodes with degree 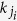 in layer *i*.

Similar to Eqs (11) and (12), we can get the critical threshold


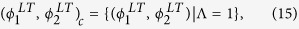


and the size of giant component


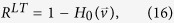


where 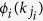 is defined as Eq. (13), for layer node-based targeted attack on multiplex networks consisting of two network layers.

**Figure 5:**
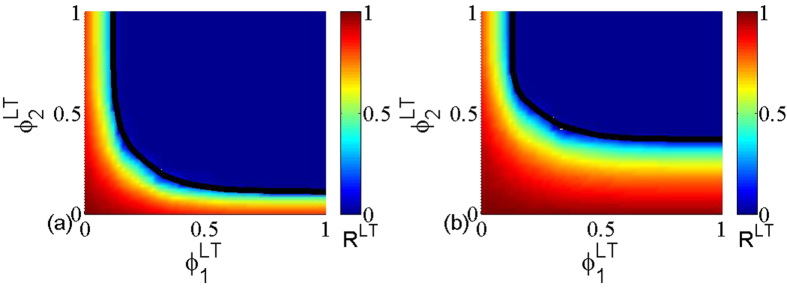
The size *R*^*LT*^ of giant component in dependence on removal probability 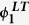 and 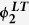 for layer node-based targeted attack. The black line indicates the theoretical critical threshold calculated according to Eq. (15). The networks used are multiplex ER networks with average degree (**a**) *z*_1_ = *z*_2_ = 2, (**b**) *z*_1_ = 2, *z*_2_ = 4 and network size *N* = 5000.

**Figure 6:**
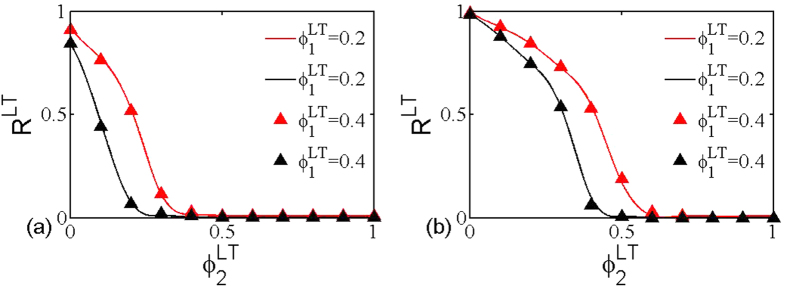
Theoretical (line) and numerical (point) results of the size *R*^*LT*^ of giant component as a function of 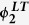 when 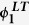 takes fixed values. The networks used are multiplex ER networks with average degree (**a**) *z*_1_ = *z*_2_ = 2, (**b**) *z*_1_ = 2, *z*_2_ = 4 and network size *N* = 5000.

In Fig. 5, the color code represents the size *R*^*LT*^ of the giant component as a function of the removal probability 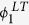 and 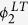 under layer node-based targeted attack, and the black line indicates the theoretical critical threshold calculated according to Eq. (15). Similar to Fig. 3, the theoretical prediction fully agrees with the simulation results. Moreover, Fig. 6 provides the further comparison between the theoretical prediction and simulation for the size of giant component, which also validates the accuracy of theoretical method. Combining with all the above phenomena, it is clear that the proposed theoretical framework can allow us to accurately calculate the critical threshold and the size of giant component under the layer node-based attack.

### Comparison of robustness of multiplex networks

Based on the above framework, multiplex node-based attack proposed in^44^, can be regarded as a special case of layer node-based attack when all the removed nodes or replicas are the same in each network layer. From the economic viewpoint, the cost of removing *p* fraction of multiplex nodes seems approximately equal to that of removing *p* fraction of layer nodes in each network layer. However, the damage of both scenarios on the multiplex networks may be greatly different. In this sense, it becomes very instructive to compare the robustness of multiplex networks under multiplex node-based attack and layer node-based attack. For simplicity of comparison, we assume that layer node-based attack means to remove the same proportion of layer nodes in each network layer in what follows. The removal probability correspondingly becomes 
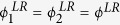
 for layer node-based random attack and 
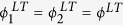
 for layer node-based targeted attack. While for multiplex node-based attack, the total fraction of removal multiplex nodes under random attack and targeted attack becomes 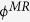 (all of the multiplex nodes are removed randomly with probability 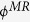) and


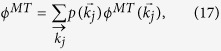


where 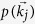 indicates the fraction of multiplex nodes with degree 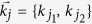, and 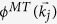 is defined as the removal probability of multiplex nodes with degree 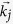 and given by


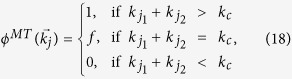


where *k*_*c*_ is the cutoff degree and *f* denotes the removal probability of node which satisfies 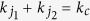.

Similar to the above treatment, we still use the critical threshold as a uniform evaluation index for multiplex node-based attack and layer node-based attack. In fact, the larger the value of critical threshold, the better the robustness of multiplex networks against attack. To get a more intuitive comparison, we consider the simple case of random attack on multiplex ER networks with *z*_1_ = *z*_2_ = *z*. Based on Eq. (9), we have 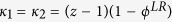 and 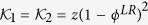. The critical threshold of layer node-based random attack 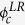 thus becomes


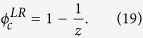


Similarly, according to ref. 44, the critical threshold of multiplex node-based random attack 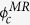 is


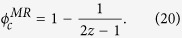


**Figure 7:**
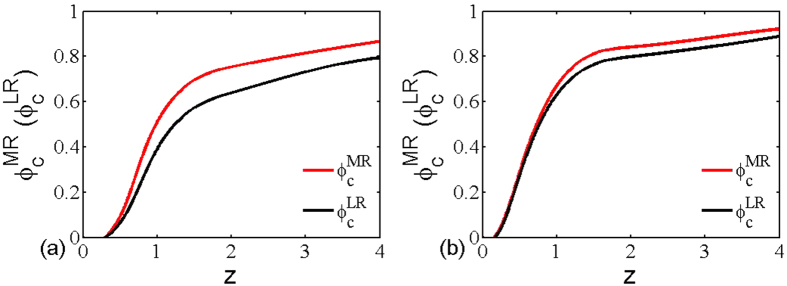
The critical threshold of multiplex networks in dependence on the network average degree under multiplex node-based random attack (red dash line) and layer node-based random attack (black solid line). The networks used are (**a**) multiplex ER networks with average degree *z*_1_ = *z*_2_ = *z* and (**b**) multiplex SF networks with average degree *z*_1_ = *z*_2_ = *z*. The size of all the networks is *N* = 5000.

**Figure 8:**
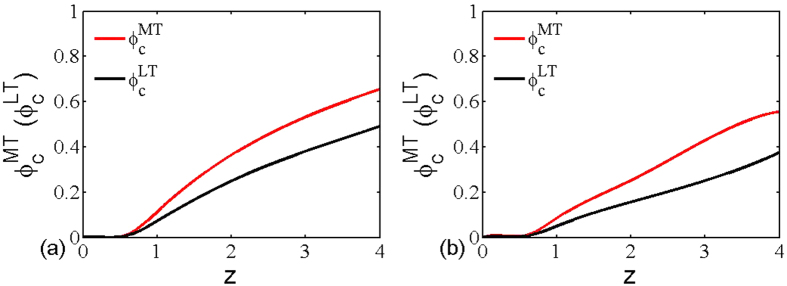
The critical threshold of multiplex networks in dependence on the network average degree under multiplex node-based targeted attack (red line) and layer node-based targeted attack (black line). The networks used are (**a**) multiplex ER networks with average degree *z*_1_ = *z*_2_ = *z* and (**b**) multiplex SF networks with average degree *z*_1_ = *z*_2_ = *z*. The size of all the networks is *N* = 5000.

Obviously, there always exists 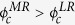, which means multiplex networks are more robustness under the multiplex node-based random attack, irrespective of average degree. To attest this theoretical analysis, we will provide more comprehensive simulation comparisons in what follows. Figure 7 features how the the critical threshold of multiplex networks varies as a function of average degree under both multiplex node-based random attack (red line) and layer node-based random attack (black line). It is clear that the threshold of both cases rises with the increment of average degree, which means that multiplex networks are more robust for denser connections. Interestingly, another observation of utmost significance is that the threshold of multiplex node-based random attack is always higher than that of layer node-based random attack, irrespective of the average degree and underlying connection topology. This computation outcome completely agrees with the aforementioned theoretical prediction. This is to say, multiplex networks are more vulnerable under layer node-based attack, because it usually makes more multiplex nodes subject to attack and lose more connections with other multiplex nodes. Moreover, we can also obtain the similar observation for multiplex node-based targeted attack and layer node-based targeted attack in Fig. 8, which further supports the fact that layer node-based attack brings larger damage to multiplex networks. Along this seminal finding, it may shed new light into the research of protection or immunization of empirical multiplex topology^53^,^54^.

## Summary

To sum, we have studied the robustness of multiplex networks under layer node-based attack. Under this framework, the layer nodes can be removed randomly or intentionally, which corresponds to layer node-based random attack or layer node-based targeted attack. A theoretical method is proposed to evaluate the robustness of multiplex networks when a fraction of layer nodes are removed. Through numerous simulations, this method can accurately calculate the threshold and size of giant component, irrespective of the removal case. In addition, we also compare the robustness of multiplex networks under multiplex node-based attack and layer node-based attack. An interesting finding is that multiplex networks will be more robust under multiplex node-based attack, which is universal for different average degree and underlying topology. With regard to the reason, it may be related with the fact that layer node-based attack usually brings damage to more multiplex nodes, which will directly break the remaining joint component of networks.

Since multiplex framework is ubiquitous in realistic social and technological networks, we hope that the present outcomes can inspire further research of the robustness of multiplex networks, especially combining with the novel properties of multiplex networks, like the clustering characteristic^25^, degree-degree correlation between network layers^37^. In addition, the targeted attack can also be incorporated into other centrality measures, such as the eigenvector centrality, *k*-shell centrality and betweenness centrality^52^. Along this line, we may get new understanding for the protection of multiplex network.

## Additional Information

**How to cite this article**: Zhao, D.-w. *et al.* The robustness of multiplex networks under layer node-based attack. *Sci. Rep.* **6**, 24304; doi: 10.1038/srep24304 (2016).

## Figures and Tables

**Figure 1:**
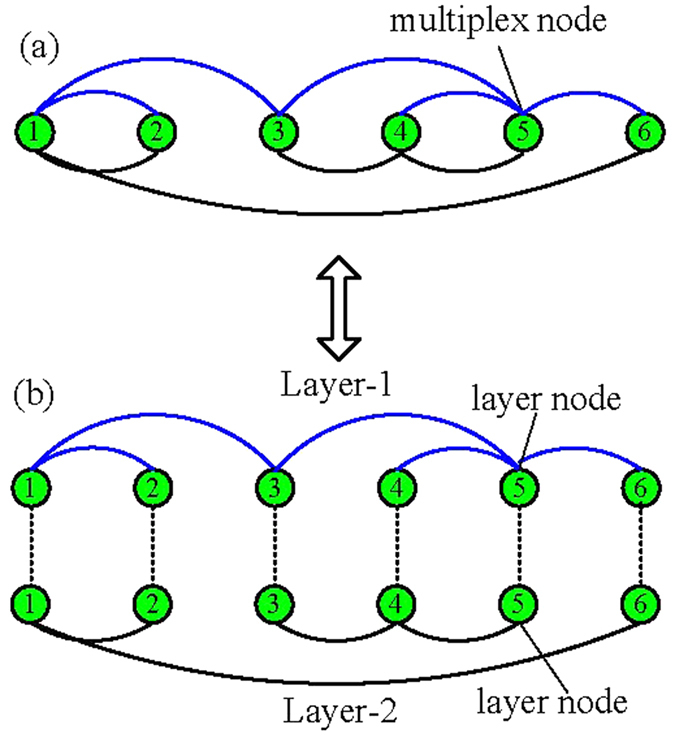
Schematic illustration of multiplex networks. (**a**) Six nodes are connected via two kinds of links (blue link and black link). (**b**) Such a system can also be embedded into the framework of multiplex networks, where each link type defines a network layer. Besides, we also define the terminology: multiplex node and layer node in the total architecture and each layer, respectively.
